# Primary leptomeningeal melanomatosis successfully treated with PD-1 inhibitor pembrolizumab: A case report

**DOI:** 10.1097/MD.0000000000022928

**Published:** 2020-12-11

**Authors:** Ana Misir Krpan, Zoran Rakusic, Davorin Herceg

**Affiliations:** a University Hospital Centre Zagreb, Kispaticeva 12; b University of Zagreb, School of Medicine; c University of Zagreb, School of Dental Medicine, Zagreb, Croatia.

**Keywords:** primary leptomeningeal melanoma, immunotherapy, pembrolizumab, case report

## Abstract

**Rationale:**

Primary leptomeningeal melanoma is an extremely rare disease of the central nervous system. There are no standard treatment protocols with a poor prognosis in very few reported cases. Immunotherapy in primary brain melanoma has not been successfully applied so far.

**Patient concerns:**

We describe a female patient 72-year-old diagnosed in the Neurosurgery Department which presented with generalized seizures.

**Diagnoses:**

Histological examination confirmed atypical melanocytes immunohistochemically positive for melan A, HMB45 and S-100 protein in the meninges, BRAF V600E negative. Dermatological, ophthalmological examinations, and 18-FDG PET/CT were negative.

**Interventions:**

The patient was successfully treated with pembrolizumab 2 mg/kg every 3 weeks for 2 years.

**Outcomes:**

The disease was stable for 2 years and the patient had no significant toxicity.

**Lessons:**

Our report describes durable intracranial tumor response suggesting the efficacy of PD-1 inhibitor pembrolizumab for central nervous system primary leptomeningeal melanoma.

## 1 Introduction

Primary leptomeningeal melanoma is a rare disease of the central nervous system representing only 1% of primary melanomas. It occurs in 1 in 10 to 20 million people.^[1]^ Given the rarity of this disease, there are no standard treatment protocols with poor prognosis in reported cases.^[2]^ Unlike the well-known facts and evidence-based treatment recommendations for cutaneous melanoma, data are lacking for the central nervous system (CNS) primary melanoma. Surgery, radiotherapy, and chemotherapy seem to be of limited benefit. BRAF V600E positive tumors can be treated with BRAF/MEK inhibitors if we interpolate treatment guidelines for brain melanoma metastases.^[3]^ The recent introduction of immunotherapy in cutaneous melanoma represents a great progression in terms of increased overall survival. Immunotherapy in primary brain melanoma has not been successfully applied so far. Below we describe a patient with primary leptomeningeal melanomatosis treated with the anti-programmed death 1 (PD-1) agent pembrolizumab.

## 2 Case report

**Figure 1:**
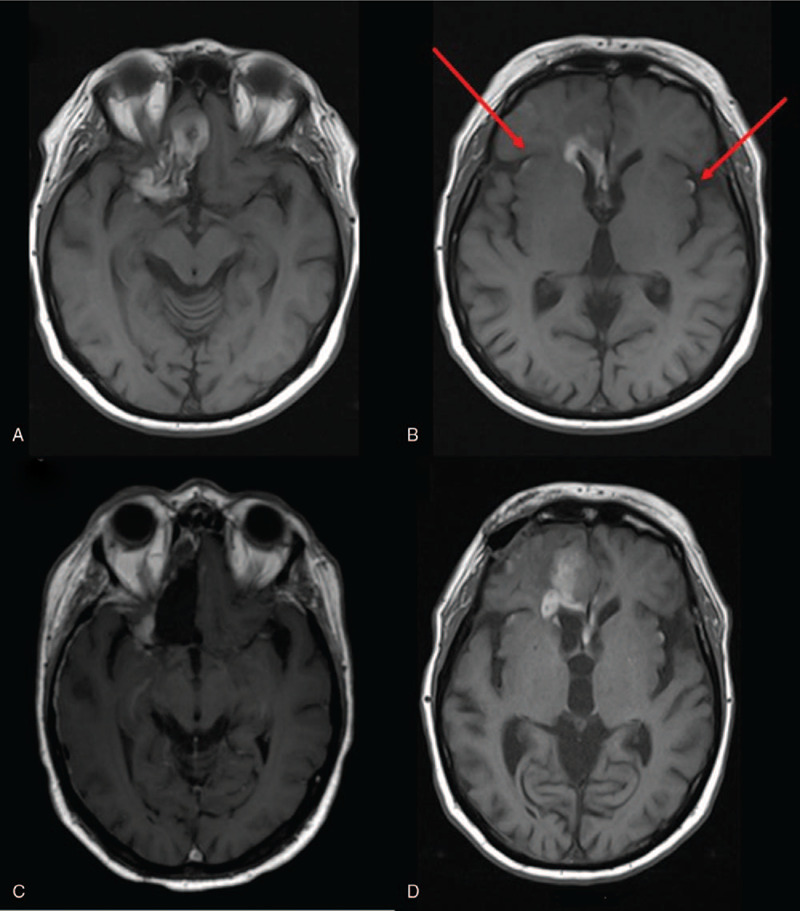
Brain MRI: on axial images hyperintense nodular tumor in right frontal lobe preoperatively in November 2016 (A) and leptomeninegeal dissemination marked with red arrows (B), postoperative residual tumor (C) and tumor progression in March 2019 (D).

A female patient, who was 72 years of age, was admitted to the Neurology Department in December 2016 with generalized seizures. She was in good general condition, ECOG 0. Contrast-enhanced brain magnetic resonance imaging (MRI) revealed an intra-axial brain tumor in the inferior gyrus of the right frontal lobe. The tumor was nodular, hyper-intense on T1 image and hypo-intense on T2 image, with heterogeneous enhancement and no significant peri-tumoral edema. The preliminary diagnosis was primary tumor (Fig. 1). The tumor was partially resected. Intraoperatively a dark brown nodular lesion in the basal part of the right frontal lobe and several leptomeningeal brownish lesions in the surrounding area macroscopically suspected for leptomeningeal melanoma dissemination were described. Histological examination confirmed atypical melanocytes immunohistochemically positive for melan A, HMB45, and S-100 protein in the meninges, BRAF V600E negative. Atypical spindle melanocytes were also present around the blood vessels and invasion of the surrounding brain parenchyma was occasionally found. The pathologist concluded that it was a melanocytic tumor, with meningeal melanomatosis consisting of diffuse and nodular subtypes. A postoperative brain MRI confirmed a residual tumor in the surgical area and leptomeningeal spread in the form of leptomeningeal thickening and avid contrast enhancement on post-contrast T1-weighted images (Fig. 1). The spine MRI was negative and lumbar puncture was not performed. Dermatological, ophthalmological examinations, and 18-FDG PET/CT were negative. The final diagnosis was primary leptomeningeal melanomatosis.

Due to leptomeningeal involvement in February 2017, the patient was treated with whole-brain radiotherapy with a total dose of 30 Gy in 10 fractions. In February 2017 pembrolizumab was started, including 2 mg/kg every 3 weeks. The patients disease was stable for 2 years without significant toxicity except for skin dryness, pruritus, and fatigue. She tolerated therapy very well, she used only the antiepileptic drug oxcarbazepine and no other concomitant medications. During the second year of treatment, discrete changes in cognitive functioning began and she complained of memory deterioration. Throughout treatment, routine laboratory findings were within normal limits, except mild eosinophilia (0.77 × 10^9^/ L) which occurred after 2 years of treatment with pembrolizumab. Although eosinophilia has been described as a sign of a good response to immunotherapy in patients with melanoma,^[4]^ we referred the patient to an infectious disease physician. The diagnosis was Strongyloidiasis, a very rare disease in our region. After a short break in therapy for the treatment of Strongyloidiasis, we continued pembrolizumab. Rare cases of Strongyloides infection have been reported in patients receiving immune checkpoint inhibitors for the treatment of melanoma. The main risk factor was administration of corticosteroids and/or infliximab.^[5]^ Our patient was not receiving corticosteroids or any additional medications except oxcarbazepine.

**Figure 2:**
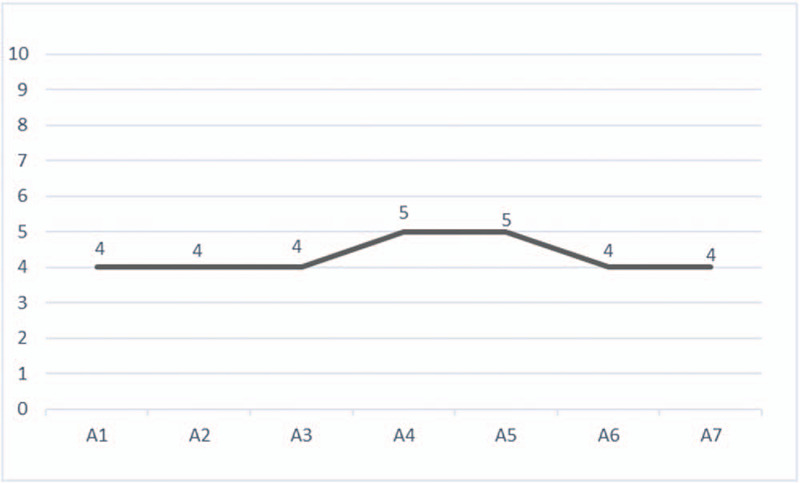
The patient's performance on AVLT 3 years after diagnosis.

In March 2019 an MRI described frontal and leptomeningeal tumor progression and mild brain atrophy (Fig. 1). She was ECOG 1 due to neurocognitive decline. A psychological test revealed a significant deterioration in time orientation, recall, concentration, calculus, drawing, and writing. During the psychological testing, the most notable feature was the slow speed of processing information. Verbal fluency was very low. An auditory verbal learning test (AVLT) showed a reduced capacity for immediate recall of words, significantly impaired learning during repetition, and reduced capacity for delayed recall (Fig. 2). The Trail Making Test (TMT) showed decreased scores. The Mini Mental State Exam (MMSE) was 17. On the emotional plan, the patient had hypomimia and emotional flatness. Currently, 3 years from the onset of the disease, she is still alive, but experience further neurological worsening due to disease progression.

## 3 Discussion

Primary leptomeningeal melanocytic neoplasms are a group of rare tumors originating from melanocytes of the leptomeninges. The diagnosis is very challenging because of nonspecific clinical, radiographic, and laboratory findings. MRI can be normal or document very discreet changes, nodular or diffuse enhancement of leptomeninges or cranial nerves. Cerebrospinal fluid analysis can remain negative despite repeated lumbar punctures. Even after obtaining a pathohistological diagnosis, additional time is required for chest-abdomen CT scan and other assessments to rule out primary extracranial melanoma. Delays in the diagnosis may influence the final outocome.^[6]^ Integrated molecular profiling is highly recommended since new biomarkers are emerging giving us an answer about biological behavior and malignant potential of the tumor as well as distinguishing primary from metastatic melanocytic tumors of the central nervous system.^[7]^ Primary leptomeningeal melanomatosis is an entity with a dismal prognosis culminating in death in several months.^[2]^ There is no consensus regarding the best treatment except for neurosurgical tumor reduction in the case of the nodular appearance of the tumor. Traditionally used adjuvant chemotherapy or radiotherapy did not show meaningful benefit.

There is evidence that immune checkpoint inhibitors pembrolizumab and nivolumab might be beneficial in patients with intracranial metastases from other primaries suggestive of blood–brain barrier permeability.^[8]^ Immunotherapy has proved to be effective in intracranial control of melanoma brain metastases, too. In the phase 2 clinical trial pembrolizumab showed activity in brain metastases in patients with melanoma with an acceptable safety profile and regardless of PD-L1 status.^[9]^ Pembrolizumab is a humanized monoclonal antibody that is directed against human cell surface receptor programmed death 1 (PD-1) with potential immune checkpoint inhibitory and antineoplastic activities. The drug is indicated for the adjuvant treatment of adults with stage III melanoma and unresectable or metastatic melanoma. At the moment there is a lack of data on the efficacy of immunotherapy in primary leptomeningeal tumors.

El Hobnouni and colleagues reported a patient treated with pembrolizumab for primary malignant melanoma of the central nervous system, but in their case, the patient failed to respond to 16 weeks of treatment with pembrolizumab which is in contrast to our durable response.^[10]^ In our patient, the clinical benefit of stable disease for 2 years was achieved with pembrolizumab.

At this moment our patient is still alive, 3 years from the onset of the disease but now with major cognitive and neurological deterioration. Significant cognitive deterioration is probably multifactorial. Because of tumor location, some symptoms like cognitive, emotional, and behavioral changes can be part of frontal lobe disorder. Patients that underwent whole-brain radiotherapy for brain metastases exhibited a significant decrease in the MMSE and other psychological tests as early as 4 months after therapy with a worsening trend during a longer period. Furthermore, described adverse effects are more pronounced in elderly patients. Whether pembrolizumab can cause cognitive impairment is not described but it is not specifically evaluated in most melanoma clinical trials. Bartels et al reported about neuronal antibodies in melanoma patients that can be associated with cognitive impairment, too.^[11]^

The limitation of this report is lacking additional genetic analysis such as looking for GNAQ, GNA11, NRAS mutations, or tumor mutation burden.

In conclusion, to the best of our knowledge, this is the only case of meningeal melanomatosis which has been successfully treated for 2 years with pembrolizumab. We achieved a durable intracranial response confirming the efficacy of pembrolizumab for CNS primary melanoma. The significance of this approach will be validated with more treated patients, thus determining the role of anti-PD-1 in this rare clinical entity.

## Acknowledgments

We thank a psychologist Ms. Ivona Poljak for a detailed psychological testing.

## Author contributions

**Conceptualization:** Ana Misir Krpan, Zoran Rakusic.

**Visualization:** Ana Misir Krpan, Davorin Herceg.

**Writing - original draft:** Ana Misir Krpan.

**Writing - review & editing:** Ana Misir Krpan, Zoran Rakusic.
